# Molecular quantification of *Plasmodium* parasite density from the blood retained in used RDTs

**DOI:** 10.1038/s41598-019-41438-0

**Published:** 2019-03-25

**Authors:** Ailie Robinson, Annette O. Busula, Julian K. Muwanguzi, Stephen J. Powers, Daniel K. Masiga, Teun Bousema, Willem Takken, Jetske G. de Boer, James G. Logan, Khalid B. Beshir, Colin J. Sutherland

**Affiliations:** 1 London School of Hygiene and Tropical Medicine, Keppel Street, London, WC1E 7HT United Kingdom; 2 Laboratory of Entomology, Wageningen University & Research, 6708 PB Wageningen, The Netherlands; 3 International Centre of Insect Physiology and Ecology, Nairobi, Kenya; 4 Computational and Analytical Sciences, Rothamsted Research, Harpenden, Hertfordshire, AL5 2JQ United Kingdom; 5 Animal Health, International Centre of Insect Physiology and Ecology, PO Box 30772-00100, Nairobi, Kenya; 6 Medical Microbiology, Radboud University Medical Centre, Geert Grooteplein 26-28, 6525 GA Nijmegen, The Netherlands; 7 Present Address: Department of Biological and Agricultural Sciences, Kaimosi Friends University College, a constituent college of Masinde Muliro University of Science and Technology, Kaimosi, Kenya; 8 Present Address: Rylands Farm, East Lambrook Road, South Petherton, Somerset, TA13 5HP United Kingdom; 9 Present Address: Netherlands Institute of Ecology, 6708 PB Wageningen, The Netherlands

## Abstract

Most malaria-endemic countries are heavily reliant upon rapid diagnostic tests (RDT) for malaria case identification and treatment. RDT previously used for malaria diagnosis can subsequently be used for molecular assays, including qualitative assessment of parasite species present or the carriage of resistance markers, because parasite DNA can be extracted from the blood inside the RDT which remains preserved on the internal components. However, the quantification of parasite density has not previously been possible from used RDT. In this study, blood samples were collected from school-age children in Western Kenya, in the form of both dried blood spots on Whatman filter paper, and the blood spot that is dropped into rapid diagnostic tests during use. Having first validated a robotic DNA extraction method, the parasite density was determined from both types of sample by duplex qPCR, and across a range of densities. The methods showed good agreement. The preservation of both parasite and human DNA on the nitrocellulose membrane inside the RDT was stable even after more than one year’s storage. This presents a useful opportunity for researchers or clinicians wishing to gain greater information about the parasite populations that are being studied, without significant investment of resources.

## Introduction

Sensitive and specific diagnosis of malaria becomes increasingly important as malaria prevalence in many regions continues to decline^1^, due to scaling-up of control interventions and improved anti-malarial chemotherapy^2^. In many regions, a decline in malaria prevalence results in a transition from *Plasmodium* infections that are predominantly characterised by high parasite density, to those of low parasite density^3^. Although the latter are harder to detect, malaria elimination programmes seek to identify all infected individuals, regardless of symptoms or parasite density, to administer anti-malarial drugs and therefore to prevent onwards transmission. The use of diagnostic tools for malaria is dependent upon the setting, but as the burden of malaria falls disproportionately upon some of the world’s poorest populations^4^, diagnosis is often constrained by resources. Current recommendations from the World Health Organisation are that malaria is diagnosed by microscopy and/or rapid diagnostic test (RDT)^1^. While microscopy can be a highly sensitive method when conducted proficiently using a thick blood film (for example, by thick film, 10 parasites per μL blood [p/μL] were detected by a lead microscopist in a reference laboratory in the UK)^5^, allowing the operator to screen many blood cells, the sensitivity and precision of this method is dependent upon the microscopist and their level or access to training^6^. Although an accepted problem, there is evidence that in some settings standards of malaria diagnostic microscopy may be declining^7–9^. Rapid diagnostic tests are composed of a plastic or cardboard cassette containing a nitrocellulose strip, on which a band of monoclonal antibodies are visualised if they bind certain *Plasmodium* proteins, set within absorptive padding. They are widely available, cheap, and easy to use. However, RDT are less sensitive than microscopy (usually with a limit of detection [LOD] of approximately 100 p/μL)^10–12^ and they cannot give a quantitative estimate of the patient’s parasite burden. Further, they cannot be used to confirm parasite clearance following treatment, as circulating *Plasmodium* proteins (e.g. histidine-rich protein-2 (HRP-2)) from recently deceased parasites can continue to indicate positivity^10,13^. Due to these drawbacks, it is recommended by the World Health Organisation (WHO) that microscopy and RDT results are routinely compared in the field^14^.

In recent years, molecular techniques for detecting parasite DNA have been applied to the blood samples that are collected during the process of routine malaria diagnosis. The first studies of this kind applied polymerase chain reaction (PCR) to Giemsa-stained blood smears used previously for diagnostic microscopy^15,16^. Since then, several studies have recognised the dried blood within used RDT as a valuable source of parasite DNA, and successfully applied molecular analysis to this template. The advantages of this are multiple: RDT are widely deployed across all malaria endemic settings, the volume of blood required is generally small (e.g. 5 µL), minimising discomfort and inconvenience to the patient, there are minimal storage requirements, and DNA amplification from the blood inside used RDT (RDT_DNA_) has repeatedly been shown to be successful up to one year post-collection^17–20^. Further, there is very little possibility of cross-contamination since blood is contained within the RDT cassette, and retrospective clinical and research studies can be undertaken where whole blood or dried blood spot samples for DNA extraction were not specifically collected. While initial studies of PCR from RDT_DNA_ focussed on proof of principle and quality assessment of the PCR result^18,21^, it is now acknowledged that extraction and amplification of parasite DNA from RDT_DNA_ can allow more in-depth molecular analysis, including parasite genotyping to identify emerging resistance or important polymorphisms including HRP-2 deletions^22^, better monitoring of the progress of malaria control strategies and studies of parasite genetic diversity^18,20^. The identification of different infecting *Plasmodium* species, and indeed mixed-species infections, has also been demonstrated^21,23^. Further, the use of such methods under different epidemiological and environmental settings has now been established, with an expanding evidence base that includes studies in French Guiana^17^, Tanzania^18^, Zanzibar^19^, Mali^20^ and the Comores^23^. Methods of DNA extraction from RDT_DNA_ have been optimised in terms of the extraction procedures themselves^17,19^, and importantly have also identified the optimal internal RDT component for targeted DNA extraction^21^, whilst accounting for possible variability according to the specific brand of RDT. However, to our knowledge, the quantification of patient parasite density based on RDT_DNA_ has not previously been demonstrated.

The objective of this study was therefore to determine *Plasmodium* parasite burden in patients by duplex quantitative PCR (qPCR) based on RDT_DNA_. We validated this parasite quantification through comparison to that calculated using a simultaneously sampled dried blood spot on Whatman filter paper (DBS_DNA_). External validation is particularly critical in the context of RDT_DNA_ because the nitrocellulose membrane that has been shown to best harbour good quality parasite DNA^21^ is nonetheless not designed to this end. In addition, as it is recognised that the advantages of RDT_DNA_ include minimal long-term storage requirements, parasite quantification data after various storage times are needed to validate these conditions.

## Results

### Validation of robotic DNA extraction

**Figure 1:**
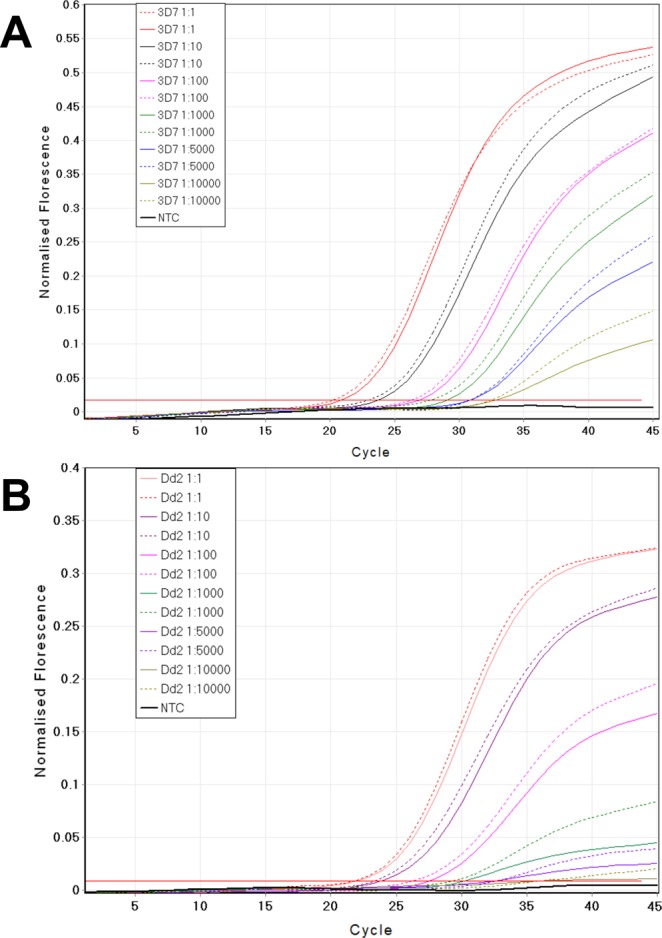
Comparison of the performance of the manual (chelex, solid lines) and automatic (QIAsymphony, dashed lines) systems for DNA extraction prior to qPCR genotyping of the pfcrt gene. (**A**) Extraction from the *P*. *falciparum* 3D7 pfcrt haplotype CVMNK, and (**B**) extraction from the *P*. *falciparum* Dd2 pfcrt haplotype CVIET. NTC is no template control.

We validated the quality of *P*. *falciparum* DNA derived from artificial dried blood spots (DBS) made with Dd2 and 3D7 cultures, by using a robotic extraction system (QIAsymphony, QIAGEN, Germany) with two different reagent and column kits from the Manufacturer (designated “Investigator” and “Blood”), in parallel with a manual (Chelex)^24,25^ extraction system. We then genotyped the extracted DNA using the commonly used *pfcrt* qPCR assay^26^. Both the manual and robotic extractions gave comparable results (Fig. 1), although the latter was slightly more efficient, exhibiting a lower average cycle threshold (CT) value compared to the former (manual 30.16 ± 5.86; automatic “Investigator” 29.37 ± 5.95; automatic “Blood”, 29.56 ± 5.96 [mean CT ± standard deviation]). Both extraction methods detected the two *pfcrt* haplotypes (CVMNK and CVIET in 3D7 and Dd2 parasites respectively) with a sensitivity as low as five parasites per microliter. The coefficient of variation (CV) at the lowest parasitaemia was 5.10% and 5.20% for “Blood” and “Investigator” kits respectively using the CVMNK probe and 5.10% and 5.12% respectively for the kits using the CVIET probe. We observed no difference in mean CT value between QIAGEN “Blood” kit and QIAGEN “Investigator” kit at the lowest parasitaemia (the latter is recommended for DNA extraction from DBS) (*P* = 0.51, paired t-test, SED = 2.4, DF = 5; Table 1), when used in the robotic system.

**Table 1:** Cycle threshold (CT) values obtained during qPCR of the pfcrt haplotypes CVMNK and CVIET in the 3D7 and Dd2 *P*. *falciparum* strains, respectively), following manual (chelex) or robotic (QIAsymphony) extraction.

Format	Method*	DNA dilution (p/μL)	3D7 CVMNK			Dd2 CVIET		
			Mean Ct	Ct STD	Ct CI	Mean Ct	Ct STD	Ct CI
Manual	Chelex	1:1 (50000)	22.86	0.34	22.58–23.14	23.10	0.28	22.87–23.33
		1:10 (5000)	25.91	0.74	25.31–26.51	25.61	0.56	25.15–26.07
		1:100 (500)	27.92	0.36	27.63–28.21	27.45	0.34	27.17–27.73
		1:1000 (50)	30.28	0.26	30.07–30.49	30.35	0.31	30.10–30.60
		1:5000 (5)	35.94	0.47	35.56–36.32	36.21	0.53	35.78–36.64
		1:10000 (1)	38.02	2.00	36.39–39.65	38.60	1.89	37.06–40.14
Automatic	Investigator	1:1 (50000)	21.8	0.30	21.56–22.04	22.72	0.49	22.32–23.12
		1:10 (5000)	25.28	0.02	25.26–25.30	26.79	0.10	26.71–26.87
		1:100 (500)	26.3	0.42	25.96–26.64	29.76	0.47	29.38–30.14
		1:1000 (50)	30.61	1.14	29.68–31.54	29.60	0.37	29.30–29.90
		1:5000 (5)	35.51	0.52	35.09–35.93	35.21	0.26	35.00–35.42
		1:10000 (1)	36.72	1.91	35.16–38.28	36.80	1.86	35.28–38.32
	Blood	1:1 (50000)	21.2	0.11	21.11–21.29	22.50	0.12	22.40–22.60
		1:10 (5000)	25.28	0.17	25.14–25.42	26.51	0.24	26.31–26.71
		1:100 (500)	27.99	0.09	27.92–28.06	29.60	0.46	29.22–29.98
		1:1000 (50)	30.75	0.09	30.68–30.82	29.62	0.23	29.43–29.81
		1:5000 (5)	35.39	0.78	34.75–36.03	35.31	0.81	34.65–35.97
		1:10000 (1)	36.72	1.91	35.16–38.28	36.40	1.86	34.88–37.92

*The robotic system was tested with two kits, “Blood” and “Investigator”, that latter being specifically recommended for use in DBS extraction.

### Correlation in parasite densities obtained in qPCR using two DNA templates

**Figure 2:**
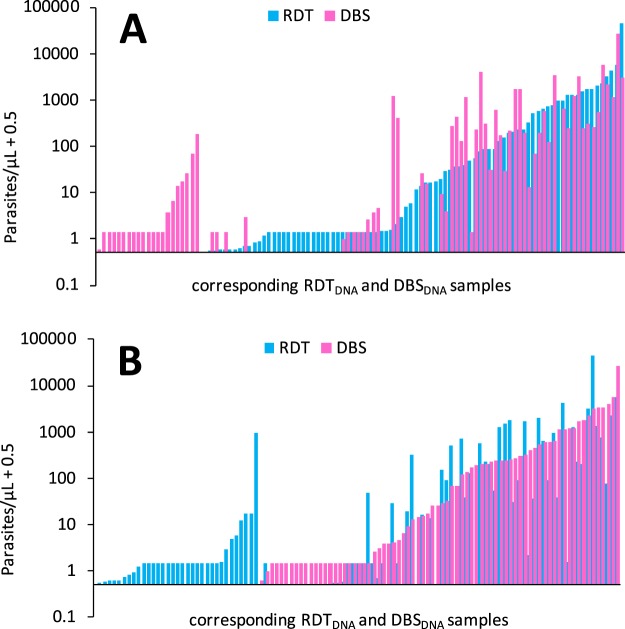
Parasite density measured by duplex qPCR, from a DNA template based on RDT_DNA_ (blue bars) or DBS_DNA_ (pink bars) (*n* = 108). Ordered by (**A**) RDT_DNA_ value, (**B**) DBS_DNA_ value (samples for which both results were zero are not shown, *n* = 33). Multiple values at 1.43 (0.93 + 0.5) p/μL represent ‘inconclusive’ samples (those that gave one positive and one negative repeat, which were universally allocated the median positive value for such samples, 0.93).

**Figure 3:**
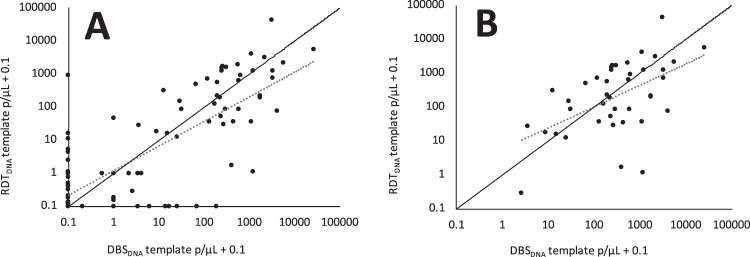
Parasite density measured by duplex qPCR, based on RDT_DNA_ or DBS_DNA_. There was a good correlation between parasite density in these paired samples across the whole dataset (**A**) (*r* = 0.78, *P* < 0.001, F = 208.77, *N* = 141), and when considering only paired samples that were both positive (**B**) (*r* = 0.53, *P* < 0.001, F = 15.41, *n* = 41). Both correlations (dashed lines) are however different from a 1:1 relationship ((**A**) *P* < 0.001, t-test; (**B**) *P* = 0.026, t-test. Data points of value 1.03 in (**A**) are missing from (**B**) as samples with one positive and one negative PCR repeat are omitted (median parasitaemia = 1.03 [0.93 + 0.1], see methods ‘Parasite quantification’).

All blood samples used in this study were RDT and filter paper finger prick samples collected in parallel from participants in a malaria odour study (described elsewhere, Supplementary Fig. S1)^27^. *Plasmodium* parasite density, quantified by duplex qPCR, was correlated across the 141 RDT_DNA_ and DBS_DNA_ paired samples (*r* = 0.78, *P* < 0.001, F = 208.77), with no difference between the parasite densities obtained by either method (Wilcoxon Matched-Pairs test z = 0.475, *P = *0.635, *N* = 141, Figs 2 and 3A). When only including pairs in which both the RDT and DBS sample was positive for parasite DNA (and both repeats of each sample), a good correlation remained (*r* = 0.53, *P* < 0.001, F = 15.41, *n* = 41, Fig. 3B) and no difference was observed between the values obtained from either method (Wilcoxon Matched-Pairs test z = −0.279, *P* = 0.781, *n* = 41). Although paired samples were only marginally different, there was a trend for the DBS_DNA_ sample to give higher parasite densities across most of the range of parasite densities (deviation from 1:1 correlation, Fig. 3A).

### Performance of templates for DNA storage and amplification

Parasite density was estimated by qPCR from one RDT_DNA_ sample to be 0.05 p/μL, with both repeats positive, suggesting a low limit of detection for this assay based on a RDT_DNA_ template. Further, given the correlation in parasite density obtained here between these two templates, and accepted ability of Whatman filter paper to preserve DNA, we deduce that storage of RDT_DNA_ for over a year (14 months: sample collection 15/05/2014–10/07/2014, first DNA extraction 28/09/2015) did not lead to DNA degradation.

We used the recovery of human DNA (*HumTuBB*) to perform an exploratory analysis, comparing the amount of DNA recovered from the two template types. By taking advantage of the within-PCR-plate normalisation of *HumTuBB* amplification CT value to the international standard (i.e. delta CT)^28^, we observed a difference between RDT and DBS sample pairs (*P* < 0.001, N = 141, Sign test), despite total surface area of the two template types available for extraction being approximately equal. A non-zero, positive median ratio of RDT:DBS delta CT values (median = 2.64, IQR: 0.83–7.67) indicated that RDT delta CT was more often a higher value, indicating a lower recovery of detectable human DNA.

### Immunochromatographic test outcome vs. qPCR parasite quantification on two templates

Approximately one third of all samples with a positive RDT test result were found to be negative by qPCR (RDT_DNA_ or DBS_DNA_), although there was not clear agreement on which samples were truly negative between the two PCR templates (Supplementary Table S1). Further, several samples yielded a negative RDT test result, were qPCR RDT_DNA_ positive or ‘inconclusive’, but qPCR DBS_DNA_ negative, indicating probable false positivity by qPCR RDT_DNA_ (Supplementary Table S1). Samples were collected from participants over three timepoints, at zero, seven and 21 days (Supplementary Fig. S1). Rapid diagnostic test positivity and qPCR negativity was disproportionately observed in timepoint two, at seven days (RDT result positive, qPCR result ‘inconclusive’ or negative: timepoint 1, qPCR RDT_DNA_ 32.3%, qPCR DBS_DNA_ 25.8%; timepoint 2, qPCR RDT_DNA_ 66.7%, qPCR DBS_DNA_ 73.3%; timepoint 3, qPCR RDT_DNA_ 29.2%, qPCR DBS_DNA_ 33.3%).

## Discussion

Duplex qPCR was successfully used to quantify parasite density in paired samples, comprising used RDTs and dried blood spots on Whatman filter paper. Despite weak evidence of higher values derived from qPCR DBS_DNA_, no statistical difference in estimated parasite density was found between the paired samples. For this analysis, we successfully validated a robotic extraction system for extracting DNA derived from dried blood spots. Lower CT values, indicating more rapid DNA amplification, were observed in qPCR assays based on robotically extracted template. This can be interpreted as improved extraction performance by the robotic system relative to the manual system and may reflect the superiority in DNA quality generated by the robotic system. Similarly, lower CT values for the human gene were observed in qPCR assays based on DBS template relative to used RDT template in the paired field samples. This, and the skew towards higher parasitaemia values derived from qPCR DBS_DNA_, may be indicative of superior DNA quality derived from the Whatman filter paper dried blood spots relative to the used RDT.

As in previous studies that compared RDT results with qPCR amplification on the same sample, a proportion (approximately one third) were found to be negative by qPCR while the RDT result was positive^18,20,21,23^. Circulating parasite antigen following anti-malarial chemotherapy would lead to this, because of continued antigen detection by RDT. Indeed, we observed this disproportionately in the second sampling timepoint, where individuals were likely to have received anti-malarial chemotherapy. As the third timepoint was 21 days after the first timepoint and 14 days after the second, the drug effect on parasite density is less likely to be observed.

A high proportion of qPCR ‘inconclusive’ samples (one positive and one negative repeat) in the RDT negative samples was probably due to the prevalence of low-density infections often found in this endemic setting^29,30^. For such samples these discrepancies are more common. As the qPCR limit of detection, estimated from duplex qPCR assays to be approximately 0.05 parasites/μL, is considerably lower than that of a standard RDT (100–200 p/μL)^10^, these samples most likely represent infections in which the parasite density fluctuates around the qPCR limit of detection. At such densities, stochastic effects can dictate whether parasite DNA is detected in the sample, in terms of the random likelihood of parasites in that particular blood aliquot, the randomness in the location of the parasites on the blood template, and the likelihood of that specific area of template being tested. Also contributing to the mismatch between RDT and qPCR results is the presence of *P*. *falciparum hrp2* and *hrp3* gene deletions, known to affect the functionality of HRP-2-based RDT, and reported elsewhere from this study cohort^22^. Of these RDT negative qPCR positive samples, there was some disagreement between RDT_DNA_ and DBS_DNA_ template qPCR results. Again, this is most simply explained by the relatively high prevalence of low-density infections in this cohort, where parasite numbers fluctuate around the point of qPCR detection and stochasticity exists in sampling.

By using a duplex qPCR that normalises parasite DNA quantification relative to the quantity of a human gene (*HumTuBB*), our assay circumvents two possible problems in the use of RDT_DNA_ as a quantitative DNA template: that the blood spot in an RDT is an imprecise volume, and that the nitrocellulose membrane was not designed to bind and store DNA and (as is suggested by our data) may do so with less efficiency than other substrates. Here, the amplification of *HumTuBB* constitutes an internal control, although poor amplification of *HumTuBB* should be identified and interpreted with caution, as this could lead to artificially raised estimates of *Plasmodium* parasite density. We used a pan-genus qPCR test with a target sequence (*pgmet* tRNA gene) that is 100% identical in nucleotide sequence in all human-infecting *Plasmodium* species^28^. Our approach would be equally effective with a duplexed qPCR test with a species-specific target for *Plasmodium falciparum*, as this is the species represented in the International Standard (INT)^31^.

In finding that the stored blood deposit within used rapid diagnostic tests presents opportunities for future molecular analysis of the *Plasmodium* parasite population, we support the findings of other studies^17–20,23,32^. We further build on this finding by demonstrating that *Plasmodium* parasite densities can be estimated by qPCR from RDT_DNA_ templates, with similar results as obtained from standard Whatman filter paper dried blood templates. This finding is of interest across a range of clinical and research settings, where in-depth retrospective molecular analysis of parasite populations can be undertaken following appropriate, minimal requirement, storage of RDT_DNA_. This study provides further evidence that the long-term storage (up to 14 months at −20 °C) of such RDT_DNA_ has no effect on the outcome of retrospective molecular assays, as previously shown^17–20^. This is in keeping with studies that suggest storage of DBS blood samples at −20 °C averts loss of assay sensitivity over time^33^, and that this is not affected by a limited number of freeze-thaw cycles, as experienced by our samples during shipment. Further, other studies have successfully amplified parasite DNA from used rapid diagnostic tests that had been stored at ambient temperature for one^18,21^, three^18^ or 14 months^20^. It would be interesting to compare parasite quantification from used RDT among different RDT cassettes/brands. Parasite detection from used RDT from 12 RDT brands was previously reported, and variable Ct values were demonstrated^21^. Therefore, the correlation between parasites densities obtained from DBS_DNA_ and RDT_DNA_ as described in the current paper may not be of similar strength to that obtained when using alternative RDT types.

In this study, rapid diagnostic tests previously used for malaria diagnosis in a cohort of 141 five- to 12-year-old children in Western Kenya were shown to provide a DNA template for *Plasmodium* parasite quantification of equal functionality to Whatman filter paper. This study further bolsters the literature indicating that rapid diagnostic tests, with adequate storage, can reliably be used for retrospective quantitative molecular studies of *Plasmodium* parasite population dynamics some months to years after original use.

## Methods

### Ethics

Five- to 12-year-old, male and female school children were recruited to a ‘malaria odour’ study^27^ after informed consent was obtained from their parent or legal guardian. The study protocol (NON SSC 389) was approved by the Scientific and Ethical Review Committee of the Kenya Medical Research Institute (KEMRI) (KEMRI/RES/7/3/1). Subsequent analyses of the blood samples were conducted at the London School of Hygiene & Tropical Medicine (LSHTM) under the ethics reference 8510. All research was performed in accordance with relevant guidelines and regulations.

### Study site and population

Participants were recruited at four schools less than 10 km from the Thomas Odhiambo Campus of the International Centre of Insect Physiology and Ecology (ICIPE) in Western Kenya (0°25′48.1″S, 34°12′24.5″E), in Suba District, Homa Bay County. In this area, community livelihoods depend upon fishing, small-scale trading or subsistence farming, and the dominant ethnic group is Luo. Malaria is endemic and transmission peaks late in the rainy season (March to August). Parasite prevalence on the nearby Rusinga island, on Lake Victoria, at this time was estimated to be 30% across the whole population^34^.

### Study design and sampling procedures

To inform sampling in the malaria odour study, participants were tested in the field for their *Plasmodium* parasite status using point-of-care methods (thick and thin blood film microscopy, and RDT [One Step malaria HRPII and pLDH antigen rapid test, SD BIOLINE, Cat no 05FK60]). Individuals who were odour-sampled were followed up at two further time-points, approximately seven (R2) and 21 (R3) days later, as described previously^27^. Repeat sampling was intrinsic to the odour study but arbitrary to this study^27^ of parasite quantification methods. At each time-point, *Plasmodium* diagnosis was repeated, and positive individuals were treated with weight-dosed artemether-lumefantrine (AL) according to manufacturer’s instructions. Throughout the entire sampling period (January–July 2014), whole blood was stored for 18S and QT-NASBA molecular assays (reported elsewhere)^27^, and RDT_DNA_ cassettes were stored for later analysis by bench-top air-drying for 24 hours, followed by storage in bundles in sealed plastic bags containing the desiccant silica gel (silicon dioxide). Between May and July, dried blood spots of approximately 5 mm were additionally collected onto Whatman No. 3 filter paper (DBS_DNA_). The latter samples provided a suitable comparator for RDT_DNA_ as a qPCR DNA template. After air-drying, filter paper samples (RDT_DNA_ and DBS_DNA_) were stored at −18 °C, other than when in transit from Kenya to London.

### Robotic extraction validation

To validate the extraction of DNA using a robotic system, we used culture-adapted 3D7 and Dd2 strains of *Plasmodium falciparum* parasites. Strains were serially diluted in blood (five-fold, starting at 1% parasitaemia) to determine the sensitivity and limit of detection of DNA extraction using both a manual (Chelex) and robotic extraction system (QIAsymphony QIAGEN, Germany), across a range of parasite densities.

Robotic DNA extraction was performed according to the manufacturer’s instructions in a deep well plate using a robotic extraction system (QIAsymphony QIAGEN, Germany), using either of two kits: “Investigator” kit (recommended for extraction of DNA from filter paper) and “Blood” kit (recommended for extraction from whole blood). Details of the procedure can be found elsewhere^22^. Manual DNA extraction for comparison was performed using the Chelex method^24^.

### Sample processing and extraction

Used RDT cassettes were opened laterally and the nitrocellulose strip was removed from inside. A central section of the nitrocellulose strip was cut out using a sterile scalpel blade and then into 1–3 small pieces of approximately 2 mm length^21^. All RDT_DNA_ nitrocellulose pieces, per sample, were extracted together. A three mm diameter circle was punched from each DBS_DNA_ using a sterile hole punch, giving a total surface area for extraction of approximately 7.07 mm^2^. Both template types were extracted using the robotic extraction system, with the QIAsymphony DSP DNA mini kit (QIAGEN, Germany) and according to the manufacturer’s instructions. In brief, buffer ATL (180 μL) and proteinase K (20 μL) were added to each well and mixed by thermomixer at 900 rpm at 560C for 15 minutes. The deep-well plate was then placed directly into the sample compartment of the QIAsymphony for DNA extraction.

### Parasite quantification

Parasite density was measured by a duplex qPCR as described previously^28^ and used previously in western Kenya^35^. This assay is designed to enable parasite quantification when the volume of blood in the sample is unknown, by normalisation against human signal. Briefly, fragments of the *Plasmodium* methionine tRNA gene and the human beta tubulin exon 4 gene (*HumTuBB*) were amplified simultaneously and detected by hydrolysis probes detected on separate fluorescent channels, allowing internal normalisation for DNA extraction efficiency. DNA extract from either RDT_DNA_ or DBS_DNA_ template (5 μL) was added to the qPCR reaction, with no-template and positive control reactions set up per PCR plate in duplicate, the latter being the INT for *P*. *falciparum* DNA^31^. Amplification assays were performed using probe-based quantitative PCR on RG3000 and RG6000 thermo-cyclers, with cycle conditions as described previously^28^. Positive samples were defined as those that crossed a pre-determined threshold of 0.025, and the cycle threshold (CT) value was taken to be the cycle number at which the amplification curve crossed this threshold. Ct values for the parasite probe fluorescence of each sample, including INT, were normalised to the *HumTuBB* gene probe fluorescence and parasite density calculated as described previously^28^. Specifically, the WHO INT DNA standard for *P*. *falciparum* comprises lyophilised blood from a hyper-parasitaemic patient who underwent exchange transfusion with an estimated parasite density pre-freezing of 4.9 × 10^5^ parasites per μL whole blood^31^. When reconstituted with 500 μL of water as the final reagent, each vial contains 500 million International Units (IU) of *P*. *falciparum* DNA. This represents 250 million parasite genome equivalents (i.e. 500 μL of human blood carrying 4.9 × 10^5^ parasites per μL), and so 1IU approximates 0.5 parasite genome equivalents, using our qPCR method.

All samples were amplified in duplicate and the average parasite density was obtained per sample. If the two repeats were positive and negative, an overall parasite density of 0.93 p/μL was assigned (median parasite density of all the positive samples in this category, across the entire malaria odour study, *n* = 71), and these samples termed ‘inconclusive’.

### Statistical analysis

We compared limit of detection of the pfcrt qPCR assay on DNA extracted using two Qiagen kits (investigator and blood). The difference in mean CT value over the different dilutions was compared using a paired t-test (*n* = 6, analyses performed in Stata [v. 15, StatCorp]). We analysed qPCR data using Rotor-Gene Q software (version 2.3.1, Qiagen), before comparing parasite densities obtained using RDT_DNA_ or DBS_DNA_ template by application of the Wilcoxon Matched Pairs Test. Quantities of DNA for RDT and DBS sample pairs were compared using the Sign test. Pearson correlations (*r*) between parasite densities from the two templates were tested using the F-test having first transformed the data using a natural logarithm with an adjustment (0.1) to allow for zero observations (analyses performed in Stata [v. 15, StatCorp]).

## Supplementary information

Supplementary Information

## Data Availability

The datasets generated during and/or analysed during the current study are available from the corresponding author on reasonable request.

## References

[1] WHO. *World Malaria Report. World Health Organization*, 10.4135/9781452276151.n221 (2016).

[2] Bhatt S (2015). The effect of malaria control on *Plasmodium falciparum* in Africa between 2000 and 2015. Nature.

[3] Okell LC, Ghani AC, Lyons E, Drakeley CJ (2009). Submicroscopic Infection in *Plasmodium falciparum* –Endemic Populations: A Systematic Review and Meta‐Analysis. J. Infect. Dis..

[4] Tusting LS (2015). The evidence for improving housing to reduce malaria: a systematic review and meta-analysis. Malar. J..

[5] Bejon P (2006). Thick blood film examination for *Plasmodium falciparum* malaria has reduced sensitivity and underestimates parasite density. Malar. J..

[6] Wongsrichanalai C (2001). Rapid diagnostic techniques for malaria control. Trends Parasitol..

[7] O’Meara WP (2005). Sources of variability in determining malaria parasite density by microscopy. Am. J. Trop. Med. Hyg..

[8] Tangpukdee N (2008). Dynamic changes in white blood cell counts in uncomplicated Plasmodium falciparum and *P*. *vivax* malaria. Parasitol. Int..

[9] Mukadi P (2016). Performance of Microscopy for the Diagnosis of Malaria and Human African Trypanosomiasis by Diagnostic Laboratories in the Democratic Republic of the Congo: Results of a Nation-Wide External Quality Assessment. Plos One.

[10] Moody A (2002). Rapid diagnostic tests for malaria parasites. Clin. Microbiol. Rev..

[11] Ochola LB, Vounatsou P, Smith T, Mabaso MLH, Newton CRJC (2006). The reliability of diagnostic techniques in the diagnosis and management of malaria in the absence of a gold standard. Lancet Infect. Dis..

[12] Wu L (2015). Comparison of diagnostics for the detection of asymptomatic *Plasmodium falciparum* infections to inform control and elimination strategies. Nature.

[13] Murray CK, Gasser RA, Magill AJ, Miller RS, Miller RS (2008). Update on rapid diagnostic testing for malaria. Clin. Microbiol. Rev..

[14] WHO. *Quality Assurance of Malaria Rapid Diagnostic Tests: Buying well and Maintaining Accuracy. World Health Organisation* (2008).

[15] Kimura M, Kaneno O, Inoue A, Ishii A, Tanabe K (1995). Amplification by polymerase chain reaction of *Plasmodium falciparum* DNA from Geimsa-stained thin blood smears. Mol. Biochem. Parasitol..

[16] Ekala MT (2000). Evaluation of a simple and rapid method of *Plasmodium falciparum* DNA extraction using thick blood smears from Gabonese patients. Bull. Soc. Pathol. Exot..

[17] Veron V, Carme B (2006). Recovery and use of *Plasmodium* DNA from malaria rapid diagnostic tests. Am. J. Trop. Med. Hyg..

[18] Ishengoma DS (2011). Using rapid diagnostic tests as source of malaria parasite DNA for molecular analyses in the era of declining malaria prevalence. Malar. J..

[19] Morris U (2013). Rapid diagnostic tests for molecular surveillance of *Plasmodium falciparum* malaria -assessment of DNA extraction methods and field applicability. Malar. J..

[20] Nabet C (2016). Analyzing Deoxyribose Nucleic Acid from Malaria Rapid Diagnostic Tests to Study *Plasmodium falciparum* Genetic Diversity in Mali. Am. J. Trop. Med. Hyg..

[21] Cnops L, Boderie M, Gillet P, Van Esbroeck M, Jacobs J (2011). Rapid diagnostic tests as a source of DNA for *Plasmodium* species-specific real-time PCR. Malar. J..

[22] Beshir KBKB (2017). Plasmodium falciparum parasites with histidine-rich protein 2 (pfhrp2) and pfhrp3 gene deletions in two endemic regions of Kenya. Sci. Rep..

[23] Papa Mze N (2016). Distribution of *Plasmodium* species on the island of Grande Comore on the basis of DNA extracted from rapid diagnostic tests. Parasite.

[24] Beshir K (2010). Amodiaquine resistance in *Plasmodium falciparum* malaria in Afghanistan is associated with the pfcrt SVMNT allele at codons 72 to 76. Antimicrob. Agents Chemother..

[25] Muwanguzi J (2016). Lack of K13 mutations in *Plasmodium falciparum* persisting after artemisinin combination therapy treatment of Kenyan children. Malar. J..

[26] Gadalla NB (2010). Dynamics of pfcrt alleles CVMNK and CVIET in chloroquine-treated Sudanese patients infected with *Plasmodium falciparum*. Malar. J..

[27] Robinson, A. *et al*. *Plasmodium*-associated changes in human odor attract mosquitoes. *Proc. Natl. Acad. Sci*. 201721610, 10.1073/PNAS.1721610115 (2018). 10.1073/pnas.1721610115 PMC5939094 29666273

[28] Beshir KB (2010). Measuring the efficacy of anti-malarial drugs *in vivo*: quantitative PCR measurement of parasite clearance. Malar. J..

[29] Okell LC (2012). Factors determining the occurrence of submicroscopic malaria infections and their relevance for control. Nat. Commun..

[30] Baidjoe AY (2016). Factors associated with high heterogeneity of malaria at fine spatial scale in the Western Kenyan highlands. Malar. J..

[31] Padley DJ, Heath AB, Sutherland C, Chiodini PL, Baylis SA (2008). Establishment of the 1st World Health Organization International Standard for *Plasmodium falciparum* DNA for nucleic acid amplification technique (NAT)-based assays. Malar. J..

[32] Papa Mze N (2015). RDTs as a source of DNA to study *Plasmodium falciparum* drug resistance in isolates from Senegal and the Comoros Islands. Malar. J..

[33] Baidjoe A (2015). The Effect of Storage and Extraction Methods on Amplification of *Plasmodium falciparum* DNA from Dried Blood Spots. Am. J. Trop. Med. Hyg..

[34] Homan T (2016). The effect of mass mosquito trapping on malaria transmission and disease burden (SolarMal): a stepped-wedge cluster-randomised trial. Lancet.

[35] Beshir K (2013). Residual *Plasmodium falciparum* parasitemia in Kenyan children after artemisinin-combination therapy is associated with increased transmission to mosquitoes and parasite recurrence. J. Infect. Dis..

