# Supplementary material for: Molecular quantification of Plasmodium parasite density from the blood retained in used RDTs
Source: Sci Rep. 2019 Mar 25;9:5107. doi: 10.1038/s41598-019-41438-0 (PMC6434039; doi:10.1038/s41598-019-41438-0)
Supplement: Supplementary file 1 — Supplementary Information [file 41598_2019_41438_MOESM1_ESM.pdf]

Molecular quantification of *Plasmodium* parasite density from the blood retained in used RDTs

Ailie Robinson*
London School of Hygiene and Tropical Medicine, Keppel Street, London, WC1E 7HT, United Kingdom.
ailie.robinson@lshtm.ac.uk

Annette O Busula
Affiliations for this study: Laboratory of Entomology, Wageningen University & Research, 6708 PB, Wageningen, The Netherlands, International Centre of Insect Physiology and Ecology, Nairobi, Kenya. Current affiliation: Department of Biological and Agricultural Sciences, Kaimosi Friends University College, a constituent college of Masinde Muliro University of Science and Technology, Kaimosi, Kenya.
abusula@kafuco.ac.ke

Julian K. Muwanguzi
London School of Hygiene & Tropical Medicine, Keppel Street, London, WC1E 7HT, United Kingdom.
Julian.Muwanguzi@lshtm.ac.uk

Stephen J. Powers
Affiliations for this study: Computational and Analytical Sciences, Rothamsted Research, Harpenden, Hertfordshire, AL5 2JQ, United Kingdom. Current affiliation: Rylands Farm, East Lambrook Road, South Petherton, Somerset, TA13 5HP, United Kingdom.
powerssj@gmail.com

Daniel K. Masiga
Animal Health, International Centre of Insect Physiology and Ecology, PO Box 30772-00100, Nairobi, Kenya.
dmasiga@icipe.org

Teun Bousema
London School of Hygiene & Tropical Medicine, Keppel Street, London, WC1E 7HT, United Kingdom, and Medical Microbiology, Radboud University Medical Centre, Geert Grooteplein 26-28, 6525 GA Nijmegen, The Netherlands.
Teun.Bousema@radboudumc.nl

Willem Takken,
Laboratory of Entomology, Wageningen University & Research, PO Box 16, 6700 AA, Wageningen, The Netherlands.
willem.takken@wur.nl

Jetske G de Boer

Affiliation for this study: Laboratory of Entomology, Wageningen University & Research, 6708 PB, Wageningen, The Netherlands. Current affiliation: Netherlands Institute of Ecology, 6708 PB, Wageningen, The Netherlands.
J.deBoer@nioo.knaw.nl

James G. Logan
London School of Hygiene & Tropical Medicine, Keppel Street, London, WC1E 7HT, United Kingdom.
James.Logan@lshtm.ac.uk

Khalid B. Beshir
London School of Hygiene & Tropical Medicine, Keppel Street, London, WC1E 7HT, United Kingdom.
Khalid.Beshir@lshtm.ac.uk

Colin J. Sutherland
London School of Hygiene & Tropical Medicine, Keppel Street, London, WC1E 7HT, United Kingdom.
Colin.Sutherland@lshtm.ac.uk

*Corresponding author

Supplementary Information

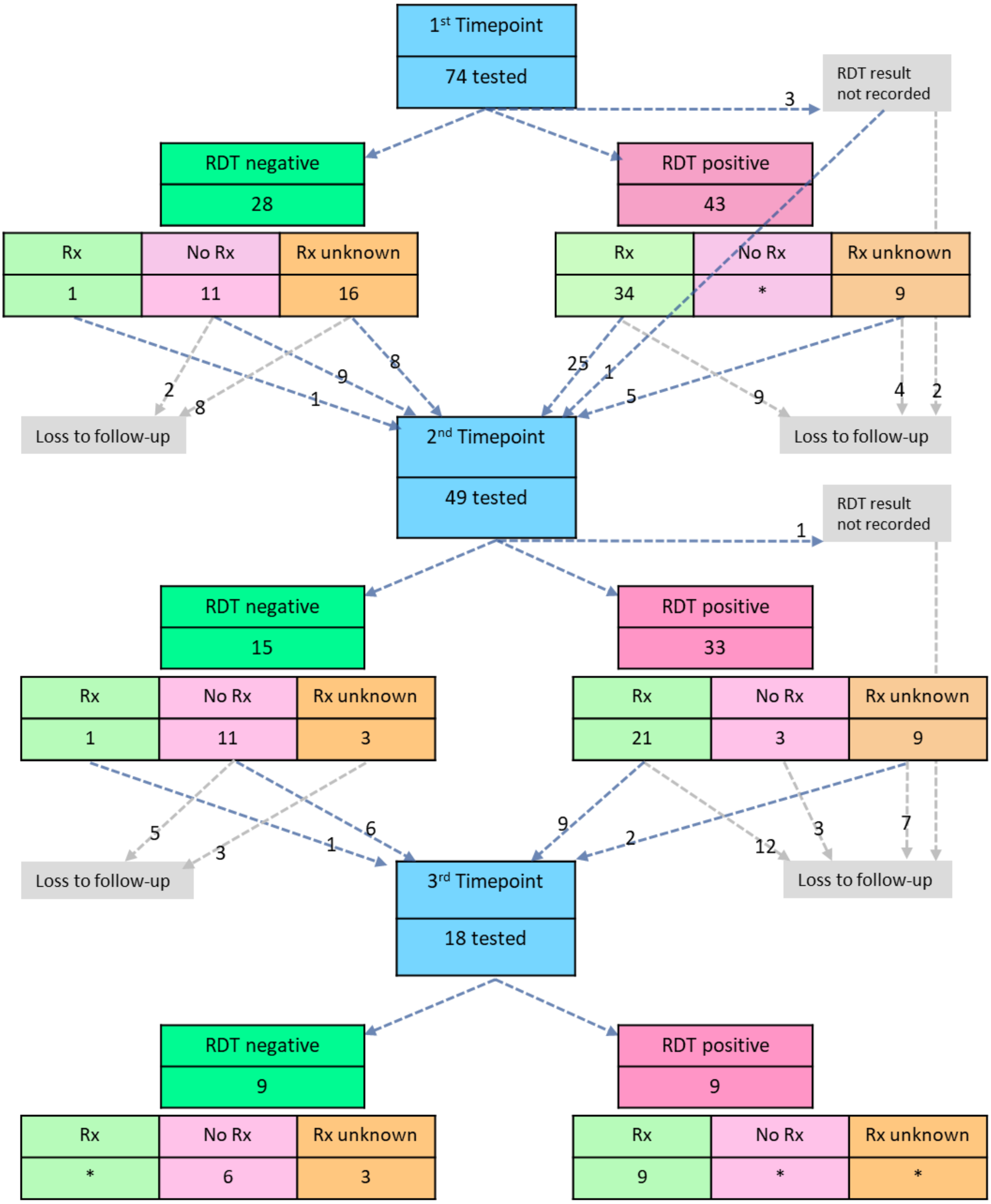

Supplementary Figure S1. The samples that were used in this study were collected from five- to 12-year-old-children, who were participating in a malaria odour study. Samples for that study were collected over three timepoints; in the present study parasite density was estimated using paired RDT and DBS blood samples from all available samples. Antimalarial treatment with artemether-lumefantrine was administered to all *Plasmodium*-positive individuals according to manufacturer's instructions.

Supplementary Table S1. The result of the RDT test (Pos P.f. or Pos P.f./pan grouped together as 'positive') in comparison to *Plasmodium* parasite positivity as determined by duplex qPCR based on two templates, fragments of the nitrocellulose membrane within a used rapid diagnostic test (RDT$_{DNA}$) or dried blood spots collected onto Whatman filter paper Number 3 (DBS$_{DNA}$). Column (1) shows the result of the RDT, columns (2) and (4) show agreement of the duplex qPCR (positive/negative) with RDT result according to the two templates, column (3) shows agreement of DBS$_{DNA}$ template qPCR with RDT$_{DNA}$ template qPCR, per RDT outcome.

**1**	**2**	**3**	**4**
RDT result, *n* (%)	qPCR RDT$_{DNA}$ template agreement with RDT result *n* (%)	DBS$_{DNA}$ template agreement with RDT$_{DNA}$ template, per RDT result *n* (%)	qPCR DBS$_{DNA}$ template agreement with RDT result *n* (%)
Positive: 86 (60.99)	Positive, 48 (55.81) [(A)]	Positive, 39 (81.25)	Positive, 47 (54.65)
		Inconclusive, 1 (2.08) [(B)]	
		Negative, 8 (16.67)	
	Inconclusive, 9 (10.47)	Positive, 3 (33.33)	Inconclusive, 9 (10.47)
		Inconclusive, 1 (11.11)	
		Negative, 5 (55.56)	
	Negative, 29 (33.72)	Negative, 17 (58.62)	Negative, 30 (34.88)
		Inconclusive, 7 (24.14)	
		Positive, 5 (17.24)	
Negative: 52 (36.88)	Negative, 23 (44.23)	Positive, 3 (13.04) [(C)]	Negative, 35 (67.31)
		Inconclusive, 5 (21.74)	
		Negative, 15 (65.44)	
	Inconclusive, 13 (25.0)	Positive, 1 (7.69)	Inconclusive, 11 (21.15)
		Inconclusive, 3 (23.08)	
		Negative, 9 (69.23)	
	Positive, 16 (30.77) [(D)]	Positive, 2 (12.5) [(E)]	Positive, 6 (11.54)
		Inconclusive, 3 (18.75)	
		Negative, 11 (68.75)	
Not recorded: 3 (2.13)	Inconclusive, 1 (33.33)	Negative, 1 (100)	Negative, 2 (66.67)
	Negative, 2 (66.67)	Negative, 1 (50)	
		Inconclusive, 1 (50)	Inconclusive, 1 (33.33)

[(A)] qPCR positive samples had detectable parasite DNA in each of two technical replicates.

[(B)] Inconclusive samples were those with one positive and one negative replicate.

[(C)] Samples were 6, 25.33, 187.50 p/μL

[(D)] Mean/median: 26.73/0.02 p/μL

[(E)] Samples were 12.47 and 8.76 p/μL
